# Impact of Removing Race Coefficient from Glomerular Filtration Rate Estimation Equations on Antidiabetics Among Black Patients

**DOI:** 10.3390/pharmacy13020052

**Published:** 2025-04-02

**Authors:** Dhakrit Rungkitwattanakul, Ebony Evans, Ewanna Brown, Kent Patterson Jr., Weerachai Chaijamorn, Taniya Charoensareerat, Sanaa Belrhiti, Uzoamaka Nwaogwugwu, Constance Mere

**Affiliations:** 1 Department of Clinical and Administrative Pharmacy Science, Howard University College of Pharmacy, Washington, DC 20059, USA; 2 Howard University College of Pharmacy, Washington, DC 20059, USA; 3 Department of Pharmacy Practice, Faculty of Pharmaceutical Sciences, Chulalongkorn University, Bangkok 10330, Thailand; 4 Faculty of Pharmacy, Siam University, Bangkok 10160, Thailand; 5 Department of Pharmacy, Howard University Hospital, Washington, DC 20060, USA; 6 Division of Nephrology, Department of Medicine, Howard University College of Medicine, Washington, DC 20059, USA

**Keywords:** equation, dosing, eligibility, race, medication dosing, antidiabetics, chronic kidney disease

## Abstract

Background: In 2021, the National Kidney Foundation–American Society of Nephrology (NKF-ASN) recommended the use of the 2021 refit equation without race; however, the effect of the removal is unclear. Our research aimed to examine the implications of antidiabetic dosing and eligibility on the new 2021 equation among Black patients. Methods: This is a retrospective analysis of patients receiving care at the diabetes treatment center (DTC) of an academic medical center. Estimated glomerular filtration rates (eGFRs) based on serum creatinine were calculated using the 2009 and 2021 CKD-EPI equations. A Monte Carlo simulation was performed to create 10,000 virtual patients. Dosing simulations based on each estimate of kidney function were performed for antidiabetics based on product labeling. The proportion and percentage of patients who were eligible based on the estimates were calculated. Results: The percentages of patients ineligible for metformin based on the estimates from the 2009 and 2021 CKD-EPI equations at the DTC were comparable (8.02% and 8.36%, respectively). In our 10,000 simulated virtual patients, the percentage of ineligibility increased only by 1%. For the GFR cut points of 20 mL/min and 25 mL/min, the rates of ineligibility were similar in our cohort and simulated patients. Conclusions: The exclusion of race from the 2021 CKD-EPI equation may slightly reduce medication eligibility among Black patients.

## 1. Introduction

Assessing kidney function through estimated glomerular filtration rate (eGFR) equations plays a crucial role in clinical practice. The estimates are essential for determining appropriate dosages and eligibility for several medications, including antidiabetics [1]. Traditionally, race is used as part of the parameter to estimate kidney function [2]. The modified diet in renal disease (MDRD) equation first introduced the inclusion of race as Black vs. non-Black in 2000 [3]. This premise was suggested based on the observation that Black individuals enrolled in the study had a higher level of serum creatinine (SCr) compared with the White counterparts at any given glomerular filtration rate (GFR). To account for this and to improve the accuracy of the equation, the multiplication factor or race coefficient was added to the equation [3]. The inclusion of race for the assessment of the GFR persisted in the medical literature for over two decades after its first mention. The subsequent iterations of GFR estimation equations (Chronic Kidney Disease Epidemiology Collaboration (CKD-EPI) 2009 and 2012 versions) still utilized race as part of the GFR calculation [4,5]. Several research studies failed to establish the benefits of using a race coefficient, rather showing the inaccuracy of GFR estimation among Black populations [6,7,8]. Therefore, in 2021, the National Kidney Foundation (NKF) and the American Society of Nephrology (ASN) formed a taskforce to address this issue and create a new 2021 CKD-EPI equation [9]. The difference between the 2009 and 2021 equations is that there is no race multiplication factor of 1.159 for the 2021 equation. This raises concerns over the implications on this new equation among Black individuals since the race multiplication factor is removed. While the new 2021 CKD-EPI equation was extensively validated and showed improved accuracy in estimating GFRs among Black individuals, the effect on medication dosing and eligibility is limited [5]. Our research aims to examine the implications of antidiabetic dosing and eligibility on the new 2021 eGFR estimation equation among Black patients.

## 2. Materials and Methods

### 2.1. Study Design

This was a retrospective, observational cohort study examining the impact of the new 2021 CKD-EPI equation on antidiabetic dosing eligibilities in patients receiving care at the diabetes treatment center (DTC) of an urban academic medical center. The reporting of this study followed the Strengthening the Reporting of Observational Studies in Epidemiology (STROBE) statement [10]. This study was approved by the office of regulatory research compliance at Howard University (IRB-2022-0800).

### 2.2. Study Population

Patients were identified via a consecutive review of medical records at the DTC in Washington, D.C. Eligible patients included individuals aged 18 years or older who received care at the DTC between January 2021 and December 2022 and self-identified as Black. The investigators also needed to be able to obtain the patients’ laboratory parameters. Patients were excluded for the following reasons: under the age of 18 years old, over the age of 90 years old, incarceration, pregnancy, received medications that alter SCr concentrations (including sulfamethoxazole/trimethoprim, cimetidine, or dolutegravir) within 7 days before data collection, or unavailable laboratory results.

### 2.3. Data Collection

Data were collected on all eligible patients using electronic medical records from 1 March 2023 to 31 October 2023. We collected demographics and required data at the time of chart access at the last visit to the DTC. Data collection included age, gender, race, weight, height, and SCr values. Estimated creatinine clearance (eCrCl) was calculated by the Cockcroft–Gault equation with various weight descriptors (total, ideal, and adjusted body weights) [11,12]. The eGFR was calculated using both the 2009 and 2021 CKD-EPI equations [4,13]. Body surface area was calculated based on weight and height and was used to calculate the de-indexed GFR [14]. The eGFR was de-indexed (converted to actual mL/min) by multiplying the automated eGFR by the patient’s body surface area (m^2^) and then dividing by 1.73 [14,15]. The decision to de-index eGFR values was made because drug clearance is proportional to the patient’s specific GFR (mL/min) rather than standardized BSA (1.73 m^2^). The original BSA standardization was derived from average American body sizes when the equation was developed, but it does not accurately reflect individuals with significantly different body sizes, such as those who are obese or severely underweight. Adjusting the eGFR to the patient’s actual BSA removes this standardization, yielding a value that better approximates the true kidney function. This adjustment is particularly relevant for obese patients, as it generally increases the eGFR, potentially moving the value above clinical thresholds that determine medication dosing eligibility. As a result, we decided to calculate the eGFR in both mL/min/1.73 m^2^ and in mL/min.

### 2.4. Study Outcomes

The primary objective was to examine the eligibility of the patients to receive antidiabetics that require dosage adjustments based on the calculated eGFR or eCrCl from different equations. The antidiabetics included metformin, glucagon-like peptide-1 receptor agonists (GLP-1RAs), dipeptidyl peptidase-4 inhibitors (DPP-4is), sulfonylureas, finerenone, and sodium-glucose cotransporter 2 inhibitors (SGLT2is). To be classified as “eligible”, the patients must have had an eCrCl or eGFR greater than the kidney function cutoffs for dosing based on product labeling approved by the United States Food and Drug Administration (US FDA) [16].

### 2.5. Statistical Analysis

Descriptive statistics were used to report patient characteristics. Categorical data were reported as frequencies with percentages and continuous data were reported as means with standard deviations or medians with interquartile ranges (IQRs). The Shapiro–Wilk test was used to evaluate the normality of the data. All analyses were performed with Microsoft Excel Version 2502 (Microsoft Corporation, Redmond, WA, USA).

To improve generalizability and robustness, we also employed the Monte Carlo simulation technique (Crystal Ball Classroom edition, Oracle) to generate 10,000 virtual patients to evaluate dosing eligibility. Data on the body weight, height, SCr, and gender of the patient obtained from the DTC were used as parameters to simulate the virtual patients. To generate a virtual patient population that accurately reflects real-world variability, we selected key parameters that influence kidney function, including age, sex, weight, height, and serum creatinine. These variables were chosen because they directly impact the calculation of the estimated glomerular filtration rate (eGFR) and subsequent drug dosing decisions. To ensure realism in the simulated population, we utilized retrospective data from our diabetes treatment center (DTC) cohort to calculate the correlation coefficient (r^2^) between body weight and height. Incorporating this correlation into the Monte Carlo simulation minimizes the creation of implausible virtual patients, such as individuals with disproportionately high body weight and short stature. By applying the observed correlation coefficient, we maintained the expected relationship between weight and height, thereby enhancing the validity of our simulated dataset. The lower limit of body weight was set at 40 kg assuming that the virtual patients are adults.

The proportion and percentage of patients (both retrospective data and simulated virtual patients) who were eligible based on each kidney function estimation equation were calculated.

To examine the agreement and bias between the eGFRs from the various equations, a Bland–Altman plot was utilized. A paired *t*-test or Wilcoxon signed-rank test was used as appropriate to determine the difference between eGFR estimates from the different equations. Cohen’s d was used to evaluate the magnitude of the difference relative to the variability of the data.

## 3. Results

### 3.1. Demographics

**Figure 1:**
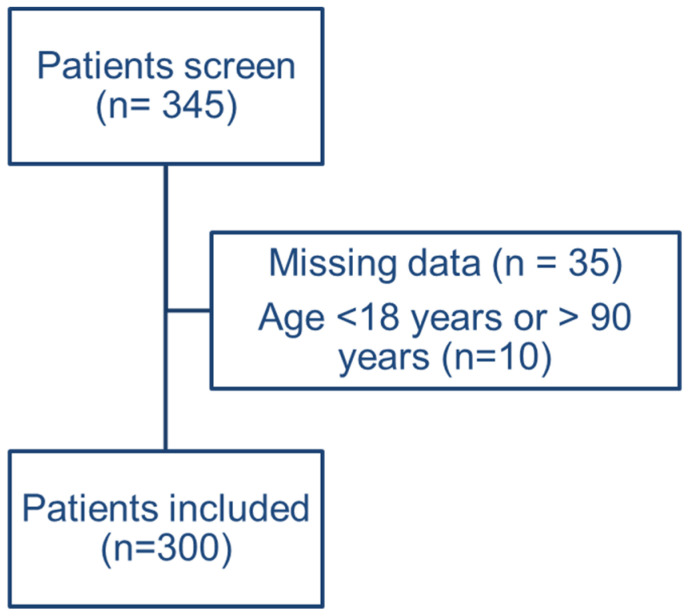
Study flow diagram.

A total of 345 patients were screened, and 300 patients were included in the study (Figure 1). Patient characteristics are included in Table 1. In the DTC cohort, the average age was 63.8 years (48–79 years). A total of 64% were female. The average BMI and BSA were 32.19 kg/m^2^ and 2.0 m^2^, respectively. The mean SCr was 1.14 mg/dL.

### 3.2. Kidney Function Assessment

Table 2 demonstrates the estimates from the various eGFR estimation equations and the Cockcroft–Gault equation among patients from the DTC and simulation groups. In the DTC cohort, patients had an average eGFR of 81 mL/min/1.73 m^2^. In the simulation group, the average was 86.7 mL/min/1.73 m^2^. Both were classified as CKD stage 2 [17]. When the estimates from the CKD-EPI 2009 and 2021 equations were adjusted with BSA, the averages were 94.1 and 86.2 mL/min, respectively.

### 3.3. Differences Between Various Estimation Equations

**Figure 2:**
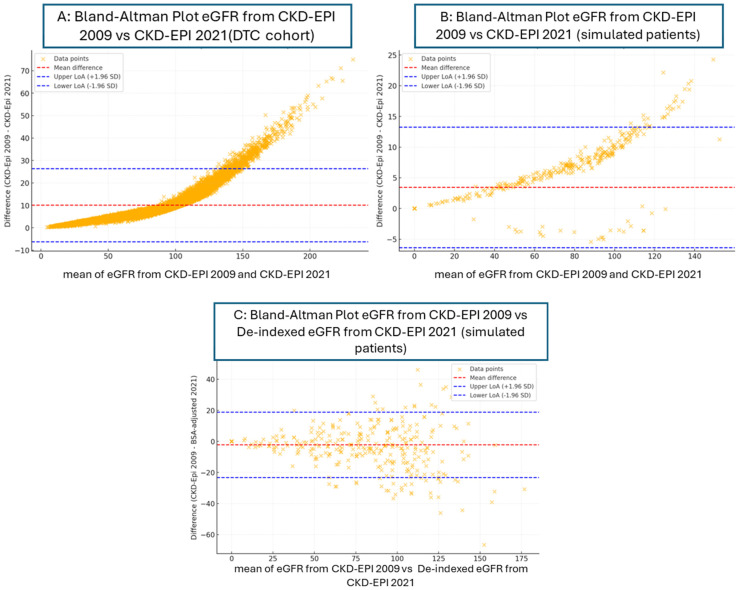
Bland–Altman plots.

As seen in Figure 2, Bland–Altman plots were used to analyze the agreements between estimates from CKD-EPI 2009 vs. 2021 and CKD-2009 vs. de-indexed eGFR from CKD-EPI 2021. The bias between estimates from CKD-EPI 2009 vs. 2021 was greater than that between estimates from CKD-2009 vs. de-indexed eGFR from CKD-EPI 2021 in both the DTC and simulated cohorts (10.06 (DTC), 3.43 (simulated) vs. 2.24) (Table 3). Paired *t*-tests were performed. Cohen’s d was measured to evaluate the magnitude of the difference between the paired data (estimated from CKD-EPI 2009 vs. CKD-EPI 2021, and CKD-2009 vs. de-indexed eGFR from CKD-EPI 2021) from the simulated cohort. There was a small magnitude of difference between estimates from CKD-2009 vs. de-indexed eGFR from CKD-EPI 2021 (Table 4).

### 3.4. Antidiabetic Dosing and Eligibility

The commonly used antidiabetic dosing recommendations in patients with kidney impairment are shown in Table 5 [16]. The antidiabetic dosing simulations and eligibilities are shown in Table 6. For medications with a GFR cut point of 50 mL/min/1.73 m^2^, there was a one-percent reduction in eligible patients from the DTC cohort when the eGFRs were calculated using CKD-EPI 2009 vs. 2021, from 80% to 79%, suggesting a modest impact. The results from the simulation cohort show similar findings, with a 3-percent decrease in the eligible patients. The eligibility rates were improved using the de-indexed CKD-EPI 2021 equation for the dosing decision. This improvement in the eligibilities of medications with various GFR cut points was found consistently in both the DTC and simulation cohorts when the de-indexed GFR from the CKD-EPI 2021 equation was utilized. The results are shown in Table 6.

## 4. Discussion

Using the 2009 CKD-EPI equation, which contains a race coefficient of 1.159, Black patients with similar demographics to White patients (gender, Scr, and age) will have a higher calculated GFR, subjecting Black patients to overestimation of the GFR [18]. Recent research aimed to reduce these gaps and disparities by modifying the 2009 CKD-EPI equation and refitting it without race [9,13], resulting in the new 2021 CKD-EPI equation. However, given that the new equation does not contain a race multiplication factor, concerns were raised given that the eGFR among Black individuals might appear lower than the estimates calculated by the 2009 version. According to our knowledge, very few studies have attempted to examine the impact of the new CKD-EPI 2021 equation on drug dosing eligibility among Black populations. In this study, we examined 300 Black patients from a DTC and amplified our data via Monte Carlo simulations to represent a larger population. The findings of our study from 300 patients from a DTC and 10,000 virtual patients showed that the impact of the new equation on drug dosing and eligibility was minimal. Moreover, the difference can be minimized by adjusting the eGFR with the patient’s specific BSA [14,15,17]. This practice is in agreement with the most updated recommendations from the American Society of Health-System Pharmacists (ASHP) on medication dosing in patients with kidney impairment [19].

Metformin is the first-line therapy for glycemic control in people with type 2 diabetes. It is safe, efficacious, inexpensive, and widely accessible. Several reports showed it may reduce cardiovascular events and mortality [20]. In large clinical trials, sodium-glucose cotransporter 2 inhibitors (SGLT2is) and a mineralocorticoid receptor antagonist—finerenone—were shown to reduce proteinuria and ultimately slow kidney disease progression to end-stage kidney disease [21,22,23,24]. Therefore, the impact of removing a race coefficient could theoretically reduce the eligibility of Black patients to access these lifesaving therapies. In our study, the 2009 CKD-EPI equation estimated eGFRs of 81.8 and 86.7 mL/min/1.72 m^2^ in the DTC and simulation cohorts, respectively. When the 2021 equation was used, the eGFRs reduced, as expected, to 75.0 and 76.6 mL/min/1.73 m^2^ in the DTC and simulation cohorts, respectively. Mean differences (bias) of 10.0 mL/min/1.73 m^2^ were found when the eGFRs from CKD-EPI 2009 vs. CKD-EPI 2021 were compared. Our findings are consistent with Miller et al. They found a decrease of 12 mL/min/1.72 m^2^ when the GFR was calculated with and without race. However, the Miller study was conducted prior to the introduction of the 2021 equation; therefore, the “without race” calculation was carried out simply by removing the race coefficient from the calculation [25]. Butrovich et al. also calculated eGFRs among Black patients and compared the estimates from the 2009 and 2021 equations. Black patients in their study exhibited a mean 10.5 mL/min/1.72 m^2^ decrease in eGFRs using 2021 CKD-EPI versus 2009 CKD-EPI [26]. The presentation of this decrease in the eGFR may position Black individuals in drug dosing ineligibility. The patients in both of our cohorts had an eGFR of 75–86 mL/min/1.73 m^2^, and both cohorts can be classified as CKD stage 2. When comparing the eGFRs from the 2009 vs. 2021 equations, 1 person was considered ineligible to receive metformin from the DTC cohort, and 196 patients (out of 10,000 virtual patients) were rendered ineligible (0.33% and 1.96% decreases in eligibility, respectively). Our results are similar to what Butrovich et al. reported, where they found similar proportions of Black and non-Black patients who were eligible to receive cisplatin between 2021 and 2009 CKD-EPI (2.7% versus 2.8%, respectively). Due to the majority of the patients in our cohort being obese (average BMI of 32 kg/m^2^, BSA of 2.0 kg/m^2^), adjusting the eGFR with BSA is recommended since drug clearance is proportional to a patient’s specific GFR and not BSA-adjusted to 1.73 m^2^ [14,15]. We found that eligibility improved when the eGFR was adjusted with the patient’s BSA. Improvements were seen consistently across the cut points.

In the 2009 CKD-EPI equation, compared with the measured GFR or true GFR, the eGFR among Black patients was overestimated by 3.7 mL/min/1.73 m^2^; on the contrary, the 2021 CKD-EPI equation underestimated the eGFR among Black individuals by 3.6 mL/min/1.73 m^2^. This shift resulted in a slight drop in the eGFR for all Black individuals when using the 2021 CKD-EPI equation vs. the 2009 CKD-EPI equation. In patients with normal or near-normal kidney function, this slight drop might not be impactful or present any clinical significance. However, for patients in CKD stage 3b or 4, a slight drop might alter the eligibility or dosage adjustment. In our simulation cohort of 10,000 patients, 117 patients were ineligible to start dapagliflozin or finerenone (1.17%), both of which are essential therapies to delay chronic kidney disease progression and proteinuria. While this is a small proportion, it is important to consider whether this new equation creates further health disparity in medicine and what we can do to mitigate this. One potential solution includes the BSA adjustment. In our study, after the adjustment, there was a 0.5% gain in eligible patients when the de-indexed eGFR from CKD-EPI 2021 was used and compared with the 2009 CKD-EPI equation. Additionally, the accuracy of eGFR measurement can be improved by the inclusion of serum cystatin C (CysC) [27,28]. The inclusion of serum CysC reduced the underestimation of 3.6 mL/min/1.73 m^2^ to 0.1 mL/min/1.73 m^2^ [13]. This could be a method to close the gap and reduce health disparities.

Our study has notable limitations. First, it is a single-center study with a relatively small sample size (300 patients from a DTC). However, we took the demographics of the population and created a set of 10,000 virtual patients to enhance the generalizability of our data. Second, patients in our cohort had mildly reduced kidney function, with the average eGFR in the CKD stage 2 range. The impact on dosing eligibility based on the new equation would be more pronounced among cohorts with more severe kidney impairment. Third, in this manuscript, we chose not to discuss the bias between the eCrCl and eGFR from the 2021 CKD-EPI equation. While we are aware that many clinicians still utilize eCrCl for decision making, it is not within the scope of the current study to determine which equation is best for drug dosing. Several professional societies recommended against the use of the Cockcroft–Gault equation for drug dosing purposes due to its inaccuracy compared to other equations [1,9,14,17,19], which made us decide not to further discuss the use of eCrCl. Forth, the dosing eligibility study was scenario-based and did not represent the true change in the clinical practice. However, we believe that our study findings are informative and demonstrate potential impacts of the 2021 CKD-EPI equation among Black populations. A major strength of our study is that our cohort comprised Black populations in a real-world setting. We were also able to amplify the data with Monte Carlo simulations to enhance the validity of our findings. Lastly, it is important to acknowledge a fundamental limitation of the MCS methodology, namely that the validity of its output is inherently dependent on the representativeness of the input data. In our study, the virtual patient population generated by the simulation was derived from local cohort data from our diabetes treatment center, which means that the findings and recommendations from our analysis are most applicable to clinical practices that share similar demographic and clinical characteristics (as shown in Table 1). MCS inherently reproduces the variability and relationships observed within the input data, but it does not inherently generalize beyond that context. As such, if the patient population in a different practice or setting significantly deviates from the characteristics of our cohort (e.g., differences in age distribution or body weight, or different degrees of kidney function), our findings may not be applicable. Consequently, when applying our recommendations, clinicians should critically assess whether their own patient population aligns with the characteristics presented in our study. Future work could involve validating these findings across diverse clinical settings to enhance generalizability and identify any potential variability in outcomes in a larger-scale real-world setting.

## 5. Conclusions

Our study shows that the adoption of the 2021 CKD-EPI equation might slightly reduce drug dosing eligibility among Black patients compared to the 2009 CKD-EPI equation.

## Figures and Tables

**Table 1:** Demographics.

Parameter	DTC Cohort (*n* = 300)	Simulated Patients (*n* = 10,000)
Age, years	63.8 ± 15.7	65.2 ± 12.7
Female sex, *n*	64	5000
Weight, kg	88.0 ± 25.8	89.9 ± 20.8
Height, cm	166.2 ± 11.6	186.2 ± 18.6
Body mass index, kg/m^2^	32.19 ± 12.1	33.49 ± 12.1
Body surface area, m^2^	2.0 ± 0.3	2.0 ± 0.4
SCr, mg/dL	1.14 ± 0.8	1.09 ± 0.8

Reported as *n* (%) or mean ± standard deviation. Abbreviations: kg: kilograms; cm: centimeters; mg: milligrams; dL: deciliters; SCr: serum creatinine.

**Table 2:** Kidney function estimates from various calculations.

Parameter	DTC Cohort (*n* = 300)	Simulated Patients (*n* = 10,000)	*p* Value
eCrCl (actual body weight), mL/min	93.1 ± 52.2	114.9 ± 59.2	0.09
eCrCl (ideal body weight), mL/min	63.5 ± 32.1	78.6 ± 34.9	0.008
eCrCl (adjusted body weight), mL/min	93.1 ± 52.2	114.9 ± 59.2	0.12
eGFR CKD-EPI 2009, mL/min/1.73 m^2^	81.8 ± 32.3	86.7 ± 42.5	0.52
eGFR CKD-EPI 2021, mL/min/1.73 m^2^	75.0 ± 28.7	76.6 ± 34.9	0.68
De-indexed eGFR CKD-EPI 2009, mL/min	94.1 ± 39.5	100.1 ± 52.3	0.25
De-indexed eGFR CKD-EPI 2021, mL/min	86.2 ± 35.1	88.4 ± 43.3	0.23

Reported as mean ± standard deviation. Abbreviations: mL: milliliters; min: minute; m: meter.

**Table 3:** Mean difference (bias) between estimates from different equations.

Bland–Altman Plot	eGFR from CKD-EPI 2009 vs. CKD-EPI 2021	eGFR from CKD-EPI 2009 vs. CKD-EPI 2021	eGFR from CKD-EPI 2009 vs. De-Indexed eGFR from CKD-EPI 2021
	DTC Cohort (*n* = 300)	Simulated Patients (*n* = 10,000)	Simulated Patients (*n* = 10,000)
Mean difference (bias)	10.06	3.43	−2.24
Upper limit of agreement (LoA)	26.38	13.24	18.83
Lower limit of agreement (LoA)	−6.37	−6.37	−23.31

Abbreviations: eGFR: estimated glomerular filtration rate; CKD-EPI: Chronic Kidney Disease Epidemiology Collaboration; LoA: limit of agreement.

**Table 4:** Cohen’s d and effect size.

Comparison	Mean Difference	Standard Deviation	Cohen’s d (Effect Size)	Effect Size Magnitude
Simulation Cohort (*n* = 10,000)				
eGFR from CKD-EPI 2009 vs. CKD-EPI 2021	3.43	5.0	0.685	Medium
eGFR from CKD-EPI 2009 vs. de-indexed eGFR from CKD-EPI 2021	−2.24	10.7	−0.208	Small

Abbreviations: eGFR: estimated glomerular filtration rate; CKD-EPI: Chronic Kidney Disease Epidemiology Collaboration.

**Table 5:** Antidiabetic dosing among different levels of kidney impairment [16].

Antidiabetics	Degree of Kidney Impairment and Dosing Recommendation
Sulfonylureas	
Gliclazide	Avoid use when eGFR < 40 mL/min/1.73 m^2^
Biguanides	
Metformin	eGFR < 45 mL/min/1.73 m^2^, maximum dose is 1000 mg/d eGFR < 30 mL/min/1.73 m^2^, do not continue
DPP-4 inhibitors	
Sitagliptin	GFR > 50 mL/min/1.73 m^2^: 100 mg daily GFR 30–50 mL/min/1.73 m^2^: 50 mg daily GFR < 30 mL/min/1.73 m^2^: 25 mg daily
Saxagliptin	GFR > 50 mL/min/1.73 m^2^: 5 mg daily GFR ≤ 50 mL/min/1.73 m^2^: 2.5 mg daily
Alogliptin	GFR > 50 mL/min/1.73 m^2^: 25 mg daily GFR 30–50 mL/min/1.73 m^2^: 12.5 mg daily GFR < 30 mL/min/1.73 m^2^: 6.25 mg daily
GLP-1 agonists	
Exenatide	GFR < 30 mL/min/1.73 m^2^: not recommended
Lixisenatide	eGFR < 15 mL/min/1.73 m^2^: not recommended
SGLT2 inhibitors	
Canagliflozin	eGFR 30 ≤ 60 mL/min/1.73 m^2^: max dose 100 mg once daily eGFR < 30 mL/min/1.73 m^2^: not recommended
Dapagliflozin	eGFR < 25 mL/min/1.73 m^2^: not recommended
Empagliflozin	eGFR < 30 mL/min/1.73 m^2^: not recommended
Ertugliflozin	eGFR < 45 mL/min/1.73 m^2^: not recommended
Mineralocorticoid receptor antagonist	
Finerenone	eGFR < 25 mL/minute/1.73 m^2^: not recommended

Abbreviations: eGFR: estimated glomerular filtration rate; mL: milliliters; min: minute; m: meter; mg: milligrams.

**Table 6:** Antidiabetic dosing eligibility.

GFR Cut Point (mL/min/1.73 m^2^)	Creatinine Clearance (mL/min)			CKD-EPI 2009 (mL/min/1.73 m^2^)	CKD-EPI 2021 (mL/min/1.73 m^2^)	De-Indexed CKD-EPI 2009 (mL/min)	De-Indexed CKD-EPI 2021 (mL/min)
	Actual BW	Adjusted BW	Ideal BW				
DTC cohort (*n* = 300)							
50	97.67	80.00	64.67	80.00	79.00	81.67	85.00
45	99.33	84.00	68.33	85.33	82.67	89.00	86.00
30	99.67	93.00	87.67	92.33	92.00	93.67	91.67
25	100.00	94.67	91.33	95.33	94.33	96.67	95.33
20	100.00	98.33	94.00	97.33	96.67	97.00	96.67
Simulated patients (*n* =10,000)							
50	77.02	77.02	61.11	75.59	72.13	79.94	76.79
45	80.82	80.82	66.58	79.43	76.26	83.46	80.58
30	92.10	92.10	83.95	90.97	89.01	92.76	91.42
25	95.07	95.07	89.26	94.00	92.83	95.61	94.51
20	97.29	97.29	93.88	96.71	95.86	97.57	97.00

Reported as % of patients who are eligible. Abbreviations: GFR: glomerular filtration rate; mL: milliliters; min: minute; m: meter; BW: body weight; CKD-EPI: Chronic Kidney Disease Epidemiology Collaboration.

## Data Availability

The data presented in this study are available from the corresponding author upon reasonable request.
